# Hsa_circ_0026416 promotes proliferation and migration in colorectal cancer via miR-346/NFIB axis

**DOI:** 10.1186/s12935-020-01593-1

**Published:** 2020-10-12

**Authors:** Yahang Liang, Jingbo Shi, Qingsi He, Guorui Sun, Lei Gao, Jianhong Ye, Xiaolong Tang, Hui Qu

**Affiliations:** Department of General Surgery, Qilu Hospital, Cheeloo College of Medicine, Shandong University, No. 107, West of Wenhua Street, Lixia District, Jinan, 250012 China

**Keywords:** hsa_circ_0026416, miR-346, NFIB, Colorectal cancer, Target therapy, Biomarker

## Abstract

**Background:**

Colorectal cancer (CRC) is one of the most common cancers worldwide. Circular RNAs (circRNAs), a novel class of non-coding RNAs, have been confirmed to be key regulators of many diseases. With many scholars devoted to studying the biological function and mechanism of circRNAs, their mysterious veil is gradually being revealed. In our research, we explored a new circRNA, hsa_circ_0026416, which was identified as upregulated in CRC with the largest fold change (logFC = 3.70) of the evaluated circRNAs via analysing expression profiling data by high throughput sequencing of members of the GEO dataset (GSE77661) to explore the molecular mechanisms of CRC.

**Methods:**

qRT-PCR and western blot analysis were utilized to assess the expression of hsa_circ_0026416, miR-346 and Nuclear Factor I/B (NFIB). CCK-8 and transwell assays were utilized to examine cell proliferation, migration and invasion in vitro, respectively. A luciferase reporter assay was used to verify the combination of hsa_circ_0026416, miR-346 and NFIB. A nude mouse xenograft model was also utilized to determine the role of hsa_circ_0026416 in CRC cell growth in vivo.

**Results:**

Hsa_circ_0026416 was markedly upregulated in CRC patient tissues and plasma and was a poor prognosis in CRC patients. In addition, the area under the curve (AUC) of hsa_circ_0026416 (0.767) was greater than the AUC of CEA (0.670), CA19-9 (0.592) and CA72-4 (0.575). Functionally, hsa_circ_0026416 promotes cell proliferation, migration and invasion both in vitro and in vivo. Mechanistically, hsa_circ_0026416 may function as a ceRNA via competitively absorbing miR-346 to upregulate the expression of NFIB.

**Conclusions:**

In summary, our findings demonstrate that hsa_circ_0026416 is an oncogene in CRC. Hsa_circ_0026416 promotes the progression of CRC via the miR-346/NFIB axis and may represent a potential biomarker for diagnosis and therapy in CRC.

## Background

Colorectal cancer (CRC) is one of the most common cancers worldwide, with an incidence of 1.8 million cases and 896,000 deaths in 2017 [1], ranking third in cancer incidence and second in cancer-related death for both sexes combined [2]. The 5-year relative survival rate of CRC patients is 65% but increases to 90% when colorectal cancers are diagnoses at a localized stage [3]. Although excellent progress has been made in elucidating the diagnosis, treatment and molecular mechanisms of CRC and other solid tumours in recent years, with therapies including surgery, chemoradiotherapy [4, 5], molecular therapy [6, 7] and immunotherapy [8–11], the poor prognosis of colorectal patients is still closely related to recurrence and metastasis [12, 13]. Blood-based markers in current clinical use, such as cancer antigen (CA) 19–9, cancer antigen (CA) 724 and carcinoembryonic antigen (CEA), are used to supervise and monitor response to therapy but have unsatisfactory sensitivity and specificity, ranging from 40 to 70% and 73 to 90%, respectively, making them insufficient as screening or diagnostic markers [14–16]. Therefore, there is an urgent need to identify an ideal biomarker to improve the rate of early diagnosis in colorectal cancer.

CircRNAs are novel stars of the non-coding RNA world that are abundant in mammalian cells, featuring relative stability and high tissue and cell specific expression [17–19]. In contrast to linear RNAs, circRNAs have covalently closed loop structures lacking 5′–3′ polarity and a polyadenylated tail [20]. Until now, benefiting from advances in RNA deep sequencing technology and bioinformatics, a large number of circRNAs has been detected in various cell lines and species [21]. Recent evidence suggests that circRNAs may play a crucial role in the occurrence and development of a variety of diseases, such as atherosclerosis, neurological disorders, prion diseases and cancers, by working as miRNA sponges, RNA-binding proteins or transcriptional regulators [22, 23]. Some circRNAs that are deregulated in CRC tissues or cell lines, such as hsa_circ_0000069, hsa_circ_0014717, circular BANP and ciRS-7, have been implicated in proliferation, metastasis and poor prognosis of CRC, indicating their crucial roles in this malignancy [24–27]. Nevertheless, the molecular mechanism of the involvement of circRNAs in CRC remains to be explored.

In our research, we conducted a comprehensive exploration of a new circRNA (hsa_circ_0026416). We found that hsa_circ_0026416 significantly upregulated in CRC tissues and high expression of hsa_circ_0026416 leads to poor prognosis. In addition, hsa_circ_0026416 acts as a ceRNA to regulate NFIB via competition for miR-346, facilitating CRC proliferation and metastasis. In summary, this study extends our knowledge on the biological roles and mechanisms of hsa_circ_0026416 in CRC and provides a novel biomarker for the diagnosis and treatment of CRC.

## Materials and methods

### Analysing circRNA expression profiling by high throughput sequencing

A circRNA expression profile (GSE77661) was downloaded from GEO. Analysis of colorectal-related differentially expressed circRNAs was performed using the Bioconductor Limma package in R software. The criteria for selection of differentially expressed circRNAs (DEcircRNAs) were *P*-value < 0.05 and |log2FC|> 1.

### Patients and clinical specimens

From January 2017 to November 2018, 169 pairs of CRC tissues and adjacent normal mucosa were collected from Qilu Hospital of Shandong University, China. At the same time, 212 cases of preoperative plasma samples and 64 cases of postoperative plasma samples were collected. All patients met the following inclusion criteria: received enterectomy and were confirmed by pathological diagnosis; complete clinicopathological data, including gender, age, tumour location, tumour diameter, tumour differentiations, pT stage, pN stage, distant metastasis, pTNM, lymphovascular invasion, and perineural invasion; complete follow-up information; and written informed consent. Patients were excluded if they received radiotherapy or chemotherapy before surgery, presented with other malignant disease within the past 5 years, were lost to follow-up, or exhibited incomplete clinicopathological data. After surgery, all patients were followed-up for at least 2 years, and patients with advanced CRC received chemotherapy and/or radiotherapy according to the National Comprehensive Cancer Network (NCCN) Guidelines. In addition, fresh normal plasma samples were obtained from 183 healthy people at Qilu Hospital of Shandong University in February 2017. All tissue and plasma samples were quickly frozen in liquid nitrogen after removal and stored at −80 °C until further experiments. This study was approved by the Ethics Committee of Qilu Hospital. Each study participant provided informed consent.

### Cell lines

The normal intestinal epithelial cell line (HCO), HEK297T and four human CRC cell lines (DLD-1, HCT-8, HCT-116, SW480) were purchased from the Culture Collection of Chinese Academy of Sciences (Shanghai, China). DLD-1 and HCT-8 cell lines were maintained in RPMI-1640 media (Hyclone, Logan, Utah, USA) with 10% foetal bovine serum (FBS, Clark Bioscience, California, USA). HCO, HEK293T, HCT-116 and SW480 cell lines were maintained in DMEM (Gibco, Grand Island, NY, USA) supplemented with 10% FBS (Clark Bioscience, California, USA) and cultured in a 37 °C incubator containing 5% CO_2_. After 2–3 stable generations, cells were used for subsequent experiments and analyses. Short tandem repeat (STR) DNA fingerprinting was utilized to identify all cell lines. Mycoplasma contamination was checked at least once a month using the MycoAlert Mycoplasma Detection Kit (Lonza, Walkersville, MD). No mycoplasma was detected in any of the cell lines.

### RNA extraction, reverse transcription PCR and qRT-PCR

Total RNA was extracted using TRIzol Reagent (Thermo Fisher Scientific, MA, USA), and the concentration and purity of total RNA were determined by an ultraviolet spectrophotometer (Eppendorf, Hamburg, Germany). Nuclear and cytoplasmic RNA isolation was performed using a PARIS Kit (Invitrogen, Carlsbad, MA, USA) following the manufacturer’s instructions. PrimeScript™ RT Reagent Kit with gDNA Eraser (Takara, Tokyo, Japan) was used to reverse transcribe RNA to cDNA. Real-time PCR was implemented using SYBR Premix Ex Taq (Takara, Tokyo, Japan) on a LightCycler® 2.0 Real-time PCR System (Roche, Basel, Switzerland). GAPDH was used as an internal reference gene. The results were analysed by the 2-ΔΔCt method. All primer sequences are listed in Additional file 1: Table S1.

### Plasmid construction and cell transfection

To overexpress hsa_circ_0026416, hsa_circ_0026416 cDNA (704 bp) was inserted into the PLCDH-ciR vector (BioSune, Jinan, China). Amplified from the cDNA of HCT-8 cells, full length NFIB was cloned into the pcDNA3.1 ( +) vector, full length of hsa_circ_0026416 and 3′UTR of NFIB was inserted into the pmirGLO vector. PmirGLO-mut (hsa_circ_0026416), pmirGLO-mut (NFIB 3′UTR), all miRNA mimics, the miR-346 inhibitor, NFIB si-RNA and 2′-O-Me-Modified-si-circ_0026416 were synthesized by Genepharma (Shanghai, China). Hsa_circ_0026416 si-RNA was obtained from Suzhou Ribo Life Science Co., Ltd (Suzhou, China).

For transient transfection of plasmids, HCT-8, SW480 and HEK293T cells were transfected with Lipofectamine 3000 reagent (Invitrogen, Waltham, MA, USA). Si-RNAs, miRNA mimics and the miRNA inhibitor were transfected into cells using the X-tremeGENE transfection reagent (Roche Applied Science, Indianapolis, IN, USA). All cell transfections were based on the manufacturer’s instructions. The sequences of these nucleic acids are listed in Additional file 1: Table S1.

### Transwell assay

Matrigel (BD Biosciences, New Jersey, USA) was added into the Transwell chamber, and the 24-well plates containing the chamber were placed into the cell culture incubator to accelerate the solidification of Matrigel. Twenty-four hours after transfection, 1 × 10^5^ (loss of function) HCT-8 cells and 2 × 10^5^ (loss of function) SW480 cells were suspended in 200 µl serum-free medium and placed into the upper chambers of a Transwell (Costar, NY, USA). When we performed gain of function and rescue experiments, 7 × 10^4^ HCT-8 cells and 1.3 × 10^5^ SW480 cells were seeded into the upper chambers of each Transwell. Then, we added 600 µl media containing 20% FBS into the lower well of each chamber. After incubation for 48 h, cells in the upper cavity were removed with cotton swabs, and cells that had invaded the lower surface were fixed, stained using crystal violet and imaged under a microscope at 100 × magnification (Olympus, Tokyo, Japan). When we assessed cell migration, all steps were the same as above, except that we did not add Matrigel.

### CCK-8 assay

For CCK-8 assay, 3000 HCT-8 or SW480 cells per well were cultured in 96-well plates, and each treatment featured three repeats. At 0, 24, 48, 72 and 96 h after transfection, 10 μl CCK-8 reagent (Dojindo Molecular Technology Company, Japan) was added to each well with the cell culture medium, and samples were placed back into the incubator for another 2 h. Absorbance was measured at 450 nm by a microplate reader (Eppendorf, Germany).

### Flow cytometry analysis

Cell cycle distribution was analysed by flow cytometry. Cells were harvested, washed twice with PBS and fixed in 75% ethanol at −20 °C for one hour. RNA was removed by incubating cells with RNase A (100 μg/ml, Sigma-Aldrich) at 37 °C for 30 min. Cells were then stained with propidium iodide (PI) solution (50 μg/ml, Sigma-Aldrich) at 4 °C for 30 min and analysed by flow cytometry (Beckman Coulter Gallios, California, USA).

### Luciferase reporter assay

The HEK293T cells were seeded in 24-well plates overnight and then co-transfected with pmirGLO-circ_0026416 WT or MUT and miR-346 mimics or NC. For HCT-8 and SW480 cells, they were co-transfected with pmirGLO-NFIB 3′UTR WT or MUT and miR-346 mimics or NC. After a 48-h culture, Firefly and Renilla luciferase activities were measured with the Dual-Luciferase Reporter Assay System (Promega, WI, USA) according to the manufacturer’s instructions, and Firefly luciferase activity was standardized by Renilla luciferase activity.

### Immunohistochemical scores

To assess protein expression, both the immunoreactive percentage and intensity were scored. The percentage of positive cells was graded as 0, < 5%; + 1, 5–25%; + 2, 26–50%; + 3, 51–75%; and + 4, 76–100% positive cells, and the intensity of cellular staining was scored as: 0, negative; + 1, weak; + 2, moderate; and + 3, strong. The final staining score was obtained by multiplying the two scores.

### Protein extraction and western blot

Protein extraction and western blot were performed as previously described [28]. Antibodies were as follows: NFIB (Abcam, ab186738, Cambridge, UK, 1:1000 dilution) and GAPDH (CST, #5174, MA, USA, 1:3000 dilution).

### In vivo tumorigenesis

BALB/c athymic male nude mice (4 weeks old) were obtained from Charles River Biotechnology (Beijing, China). SW480 cell suspensions (2 × 10^6^ cells in 0.1 ml PBS) were subcutaneously inoculated into the right axillary fossa of mice (n = 6 per group). 2′-O-Me-Modified-si-circ_0026416 was injected into the tumours every 3 days after tumours formed. Tumour growth was measured every 3 days for 22 days, at which time the mice were sacrificed. Tumour volume (V) was calculated as follows: V = (length diameter) × (width diameter)^2^ /2. The longest diameter did not exceed 2.0 cm, and the general condition of all mice was well throughout the experiment. All animal experiments were approved by the Animal Care and Use Committee of Shandong University, as well as the animal welfare settings [29].

### Statistical analysis

All data were analysed using GraphPad Prism 8.0 (La Jolla, USA). The efficiency of hsa_circ_0026416 in the diagnosis of CRC was evaluated by receiver operating characteristic (ROC) curve. The two groups were compared using two-tailed Student’s t-test. The relationship between the expression of hsa_circ_0026416 and clinicopathological characteristics was assessed by Chi-squared test. Univariate and multivariate Cox proportional hazard regression models were performed to analyse the effects of different clinicopathological factors on overall survival. Kaplan–Meier survival curve and log-rank test were used to determine the OS rate of CRC patients with different hsa_circ_0026416 expression levels. Data are displayed as means ± SD of three independent experiments. *P*-values < 0.05 were defined as statistically significant.

## Results

### Characterization of hsa_circ_0026416 in CRC tissues

**Fig. 1:**
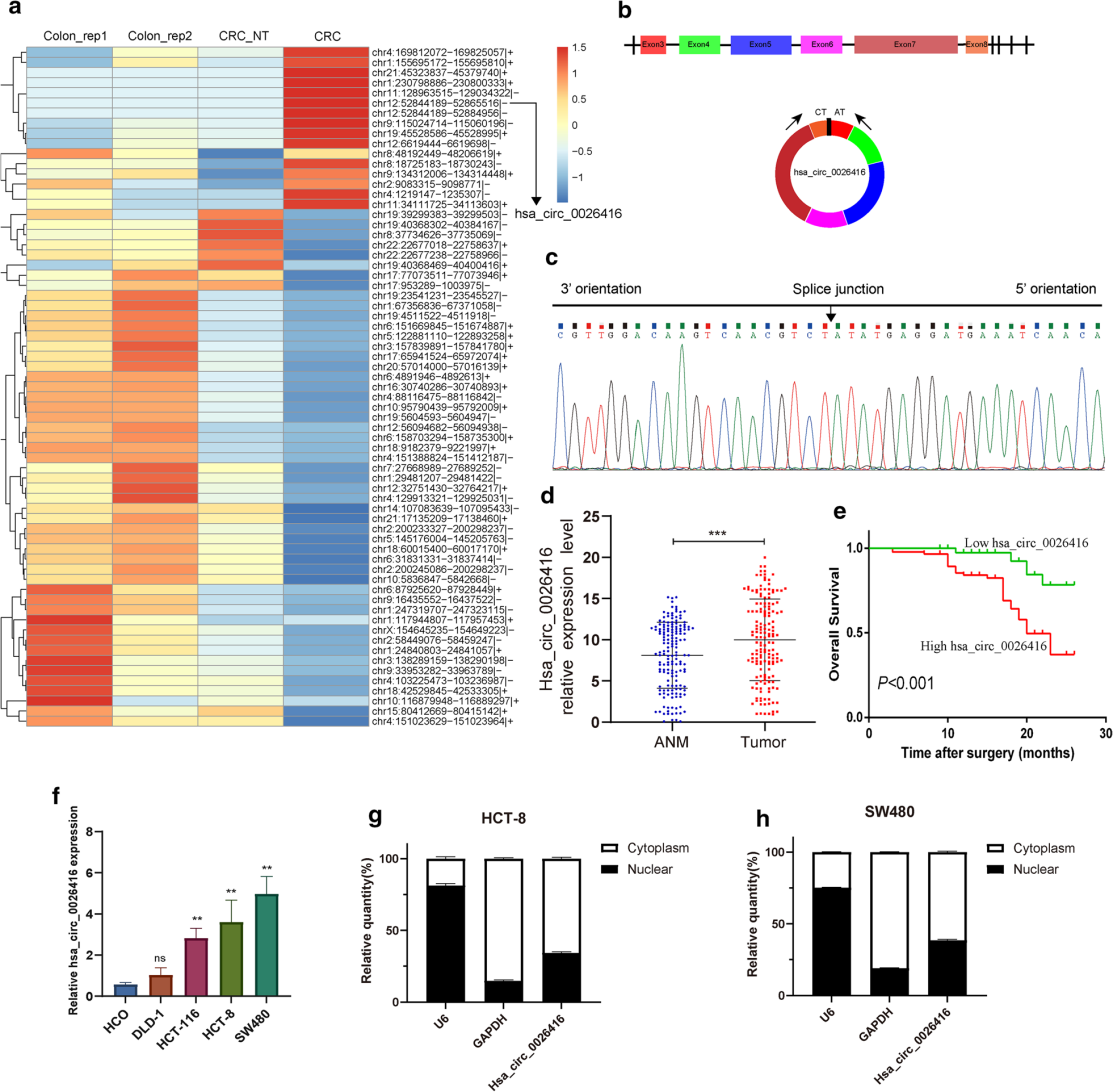
Characteristics of hsa_circ_0026416. **a** Heat map showing differentially expressed circRNAs in the GSE77661 colorectal tissue related dataset. **b** The illustration shows that the cyclization of KRT6C exons 3, 4, 5, 6, 7 and 8 form hsa_circ_0026416. **c** Sanger sequencing validates hsa_circ_0026416 splicing junction. **d** Expression levels of hsa_circ_0026416 in 169 cases of CRC tissues and their ANM using qRT-PCR. **e** Kaplan–Meier overall survival curves were created for CRC patients (n = 169) and were split into low and high hsa_circ_0026416 expression groups. **f** Hsa_circ_0026416 expression levels were detected by qRT-PCR in a normal intestinal epithelial cell line (HCO) and CRC cell lines DLD-1, HCT-116, HCT-8 and SW480. **g**, **h** qRT-PCR analysis of hsa_circ_0026416, GAPDH, and U6 in the nucleus and cytoplasm in HCT-8 and SW480 cells. Data are shown as means ± SD of three independent experiments. ns = not significant, **P < 0.01, ***P < 0.001

To explore the roles of circRNAs in the progression of colorectal cancer, we analysed colorectal tissue-related data in the GSE77661 datasets using RNA sequencing analysis. We detected 67 differentially expressed circRNAs (|logFC|> 1 and *P* < 0.05). Finally, we selected the upregulated circRNA (hsa_circ_0026416) with the largest fold change (logFC = 3.70) for additional research (Fig. 1a). Then, by querying the circBase database (https://circbase.org/), we determined that hsa_circ_0026416 is derived from regions of exons 3, 4, 5, 6, 7 and 8 of the KRT6C gene (GenBank: NM_173086) (Fig. 1b). Next, the splice junction was directly verified by Sanger sequencing (Fig. 1c). Afterwards, we detected hsa_circ_0026416 expression in CRC tissues and their corresponding adjacent normal mucosa (n = 169) by qRT-PCR. By analysing the data, we found that expression of hsa_circ_0026416 was higher in CRC tissues than in adjacent normal mucosal tissues (*P* < 0.001) (Fig. 1d). We then divided patients into two cohorts based on the median expression value of hsa_circ_0026416 (hsa_circ_0026416 high- and low-expression), and Kaplan–Meier analysis revealed that CRC patients with higher hsa_circ_0026416 expression exhibited reduced overall survival compared to CRC patients with lower hsa_circ_0026416 expression (Fig. 1e). Next, we observed that expression of hsa_circ_0026416 was higher in cancer cells of the colorectum than in normal colorectal cells (Fig. 1f). With the highest expression observed in HCT-8 and SW480, we chose these two cell lines for subsequent study. qRT-PCR analysis of nuclear and cytoplasmic RNAs indicated that hsa_circ_0026416 was primarily localized in the cytoplasm in HCT-8 and SW480 cells (Figs. 1g, h). These results revealed that hsa_circ_0026416 is an upregulated cytoplasmic circRNA and may play a vital role in CRC tumour progression.

### Hsa_circ_0026416 is a diagnostic biomarker of CRC

**Fig. 2:**
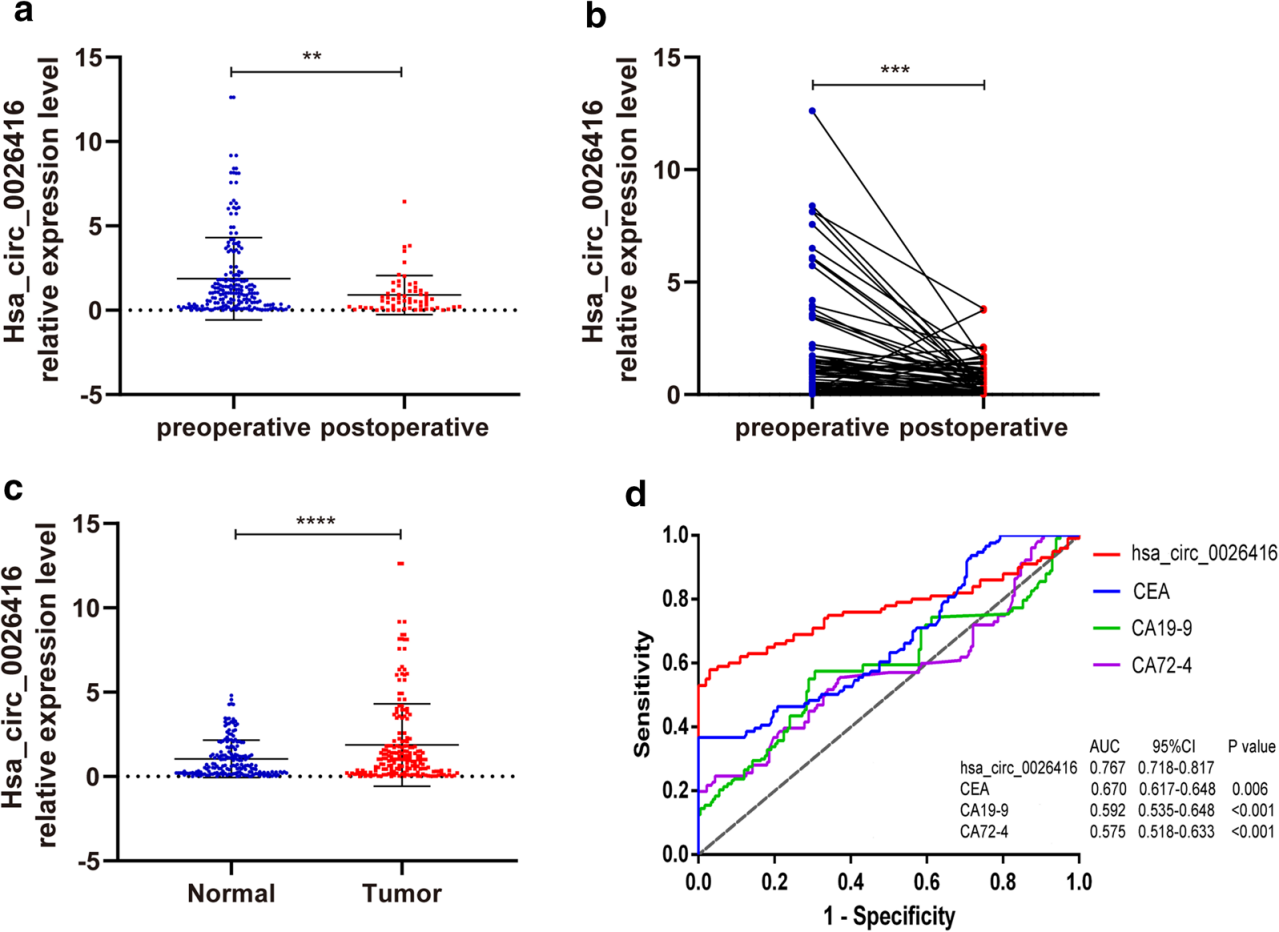
Expression of hsa_circ_0026416 in plasma. **a** Expression of hsa_circ_0026416 in CRC patient plasma before surgery (n = 212) is higher than in CRC patient plasma after surgery (n = 63). **b** Comparison of hsa_circ_0026416 expression in the plasma of 63 patients before and after surgery. **c** Expression of hsa_circ_0026416 in plasma between 212 CRC patients and 183 healthy individuals. **d** The AUC of hsa_circ_0026416 is larger than that of CEA, CA19-9 and CA72-4. Data are shown as means ± SD of three independent experiments. **P < 0.01, *** or ****P < 0.001

First, we compared hsa_circ_0026416 expression in the plasma of 212 patients with CRC before surgery with that of hsa_circ_0026416 in 63 patients after surgery (Fig. 2a, b). Meanwhile, we compared hsa_circ_0026416 expression in CRC patients’ preoperative plasma (n = 212) with healthy individuals’ plasma (n = 183) (Fig. 2c). These results demonstrated that expression of hsa_circ_0026416 in postoperative plasma was distinctly lower than in preoperative plasma (*P* < 0.01). Furthermore, compared to hsa_circ_0026416 expression in healthy individuals’ plasma, its expression in CRC patients was significantly increased (*P* < 0.001). Next, ROC curve analysis was used to assess the diagnostic ability of hsa_circ_0026416 (Fig. 2d). The AUC (area under the curve) of hsa_circ_0026416, CEA, CA19-9 and CA72-4 was 0.767, 0.670 0.592 and 0.575, respectively. To assess the correlation between hsa_circ_0026416 and clinicopathological factors, we divided patients into two cohorts according to their median expression value of hsa_circ_0026416 as a cut-off threshold (hsa_circ_0026416 high- and low-expression). We discovered that levels of hsa_circ_0026416 were significantly associated with tumour differentiation (*P* = 0.029), distant metastasis (*P* < 0.001), pTNM stage (*P* = 0.004), lymphovascular invasion (*P* = 0.002) and perineural invasion (*P* = 0.004) (Table 1). Finally, univariate and multivariate Cox regression analysis both revealed that advanced T stage, positive distant metastasis, terminal pTNM stage and high hsa_circ_0026416 expression were all indicative of poor prognosis (Table 2). These results suggested that hsa_circ_0026416 is an independent prognostic factor in CRC patients (*P* < 0.05) and has value as a biomarker for the diagnosis of CRC.

**Table 1:** Association between hsa_circ_0026416 expression and clinicopathological factors in CRC patients (n = 169)

Variables	Patients	Hsa_circ_0026416 expression		χ^2^	*P* value
	(n = 169)	High (n = 86)	Low (n = 83)		
Gender				0.00	0.992
Male	116	59 (68.6)	57 (68.67)		
Female	53	27 (31.4)	26 (31.33)		
Age (years)				0.68	0.410
≤ 60	95	51 (59.3)	44 (53.01)		
> 60	74	35 (40.7)	39 (46.99)		
Tumor location				3.45	0.063
Colon	67	40 (46.51)	27 (32.53)		
Rectum	102	46 (53.49)	56 (67.47)		
Tumor diameter (cm)				0.42	0.516
≤ 5	57	31 (36.05)	26 (31.33)		
> 5	112	55 (63.95)	57 (68.67)		
Tumor differentiation				4.78	0.029*
Poor	34	23 (26.74)	11 (13.25)		
Well / Moderate	135	63 (73.26)	72 (86.75)		
pT stage				0.30	0.582
T1–3	123	61 (70.93)	62 (74.7)		
T4	46	25 (29.07)	21 (25.3)		
pN stage				1.06	0.304
N0	93	44 (51.16)	49 (59.04)		
N1–2	76	42 (48.84)	34 (40.96)		
Distant metastasis				14.73	0.000*
M0	155	72 (83.72)	83 (100)		
M1	14	14 (16.28)	0 (0)		
pTNM stage				8.07	0.004*
I–II	73	28 (32.56)	45 (54.22)		
III–IV	96	58 (67.44)	38 (45.78)		
Lymphovascular invasion				9.51	0.002*
Yes	19	16 (18.6)	3 (3.61)		
No	150	70 (81.4)	80 (96.39)		
Perineural invasion				8.10	0.004*
Yes	8	8 (9.3)	0 (0)		
No	161	78 (90.7)	83 (100)		
Postoperative recurrence or metastasis				0.01	0.908
Yes	34	17 (19.77)	17 (20.48)		
No	135	69 (80.23)	66 (79.52)		

*Significant difference

**Table 2:** Univariate and multivariate analysis of overall survival in CRC patients

Variables	Univariate analysis		Multivariate analysis	
	HR (95% CI)	*P* value	HR (95% CI)	*P* value
Gender (male vs. female)	0.44 (0.17–1.13)	0.088		
Age (≤ 65 vs. > 65 years)	0.85 (0.43–1.68)	0.647		
Tumor diameter (≤ 5 vs. > 5 cm)	1.96 (0.99–3.85)	0.052		
Tumor location (colon vs. rectum)	1.50 (0.76–2.96)	0.240		
Differentiation (well / moderate vs. poor)	0.89 (0.39–2.06)	0.794		
pT stage (T1–T3 vs. T4)	2.02 (1.02–4.01)	0.043*	2.42 (1.14–5.12)	0.021*
pN stage (N0 vs. N1–N2)	1.60 (0.80–3.20)	0.188		
Distant metastasis (M0 vs. M1)	7.11 (3.37–15.00)	< 0.001*	4.67 (2.01–10.82)	< 0.001*
pTNM stage (I–II vs. III–IV)	5.28 (2.15–13.01)	< 0.001*	3.55 (1.38–9.16)	0.009*
Hsa_circ_0026416 expression (low vs. high)	4.59 (1.99–10.59)	< 0.001*	3.11 (1.26–7.68)	0.014*

*HR* hazard ratio, *CI* confidence interval

^*^Significant difference

### Hsa_circ_0026416 promotes CRC cell migration, invasion and proliferation in vitro

**Fig. 3:**
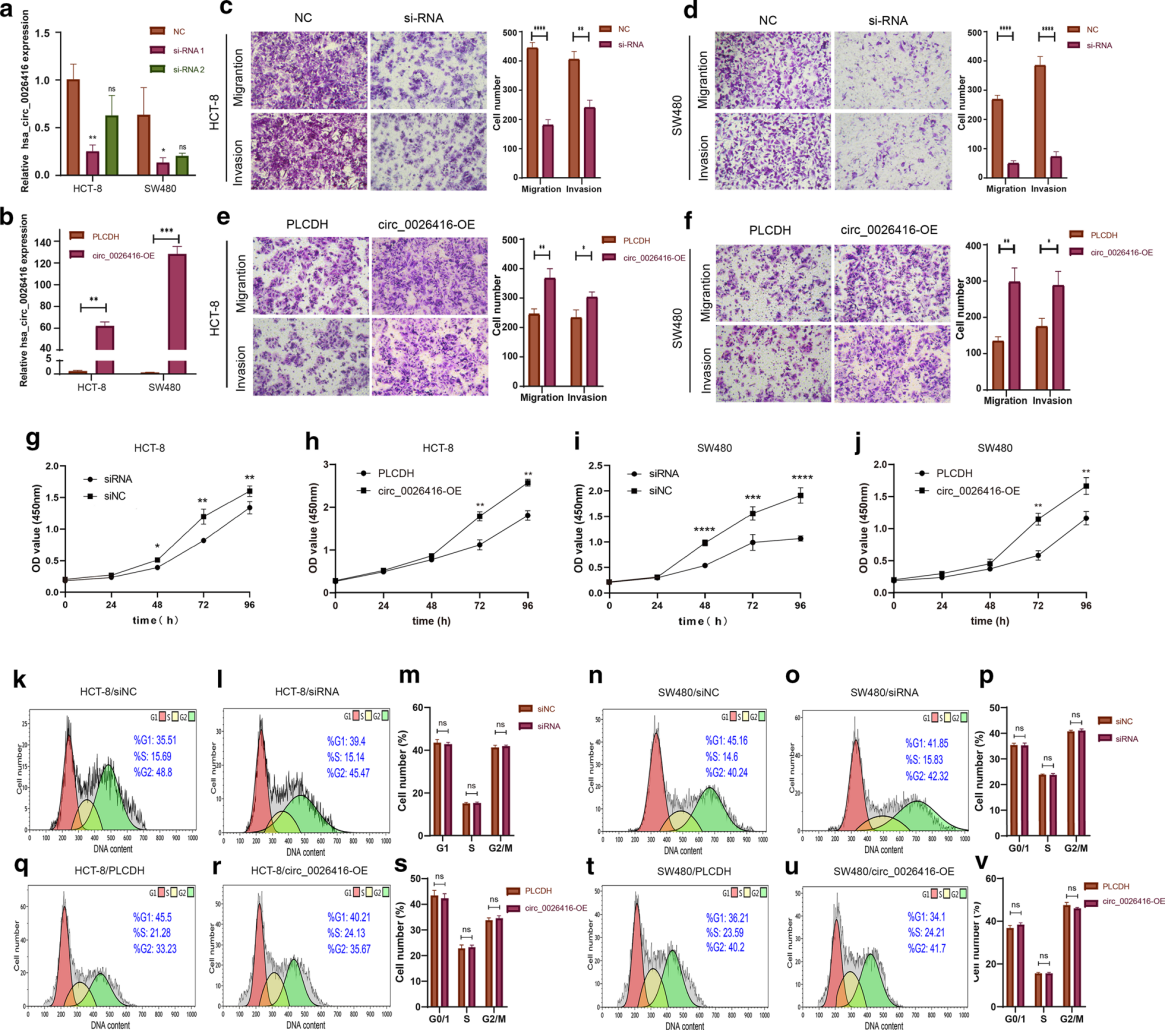
Hsa_circ_0026416 promotes CRC cell proliferation, migration and invasion in vitro. **a**, **b** Hsa_circ_0026416 was knocked down and overexpressed with transfection of an hsa_circ_0026416 overexpression plasmid and two different siRNAs in HCT-8 and SW480. **c**–**f** Transwell assays indicated that knockdown of hsa_circ_0026416 inhibited migratory and invasive ability of HCT-8 and SW480 cells, while overexpression of hsa_circ_0026416 promoted migratory and invasive capacity. **g**–**j** CCK-8 assays showing that knockdown of hsa_circ_0026416 inhibited proliferation of HCT-8 and SW480 cells, whereas overexpression of hsa_circ_0026416 enhanced proliferation capacity. **k**–**v** Hsa_circ_0026416 had no effect on cell cycle distributions in HCT-8 and SW480 cells. Data are presented as means ± SD of three independent experiments. ns = no significant, *P < 0.05, **P < 0.01, *** or ****P < 0.001

To assess the effects of hsa_circ_0026416 on colorectal cancer cell behaviour, hsa_circ_0026416 was knocked down and overexpressed by transfection with two small interfering RNAs (si-RNA1 and si-RNA2) targeting hsa_circ_0026416 and an hsa_circ_0026416 overexpression plasmid, respectively (Fig. 3a, b). Transwell assays showed that knockdown of hsa_circ_0026416 markedly inhibited the migratory and invasive capacities of CRC cells (Fig. 3c, d), while overexpression of hsa_circ_0026416 dramatically increased these abilities (Fig. 3e, f). CCK-8 assays revealed that knockdown of hsa_circ_0026416 inhibited proliferation in CRC cells (Fig. 3g, i), whereas overexpression of hsa_circ_0026416 promoted proliferation ability (Fig. 3h, j). Nevertheless, cell cycle experiments indicated that hsa_circ_0026416 had no effect on cell cycle distributions of CRC cells (Fig. 3k–v). These results prove that hsa_circ_0026416 dramatically promotes CRC cell migration, invasion and proliferation but has no effect on cell cycle distributions of CRC cells in vitro.

### Hsa_circ_0026416 acts as a ceRNA for miR-346 in CRC

**Fig. 4:**
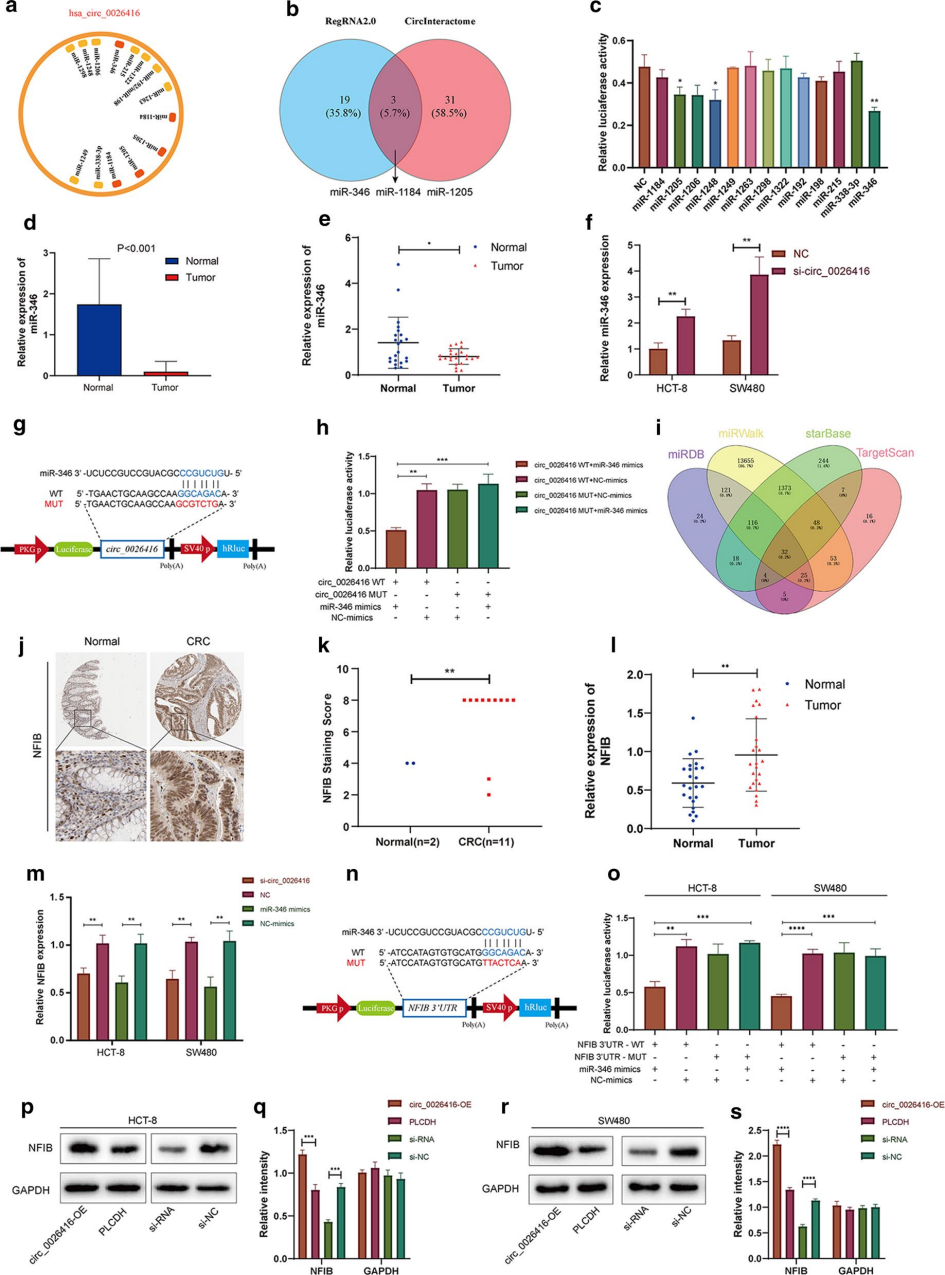
Hsa_circ_0026416 acts as a ceRNA for miR-346. **a** A schematic illustration showing the predicted binding sites of miRNAs associated with hsa_circ_0026416. **b** Venn diagram for overlaps between the two databases (RegRNA2.0 and CircInteractome). **c** Relative luciferase activity of candidate miRNAs after co-transfection of miRNA mimics and pmirGLO-circ_0026416 in HEK293T cells. **d** Expression of miR-346 in CRC tissues and ANM from TCGA data. **e** qRT-PCR for expression of miR-346 in 24 pairs of CRC and normal colorectal tissues. **f** qRT-PCR for expression of miR-346 in HCT-8 or SW480 cells after transfection of si-circ_0026416 or NC. **g** Schematic illustration of hsa_circ_0026416 pmirGLO luciferase reporter vectors. **h** Luciferase activity of WT or MUT pmirGLO-circ_0026416 after transfection with miR-346 mimics or NC in HEK293T cells. **i** Venn diagram for overlap among the four databases that predict miRNA target genes (miRDB, miRWalk, starBase v3.0, TargetScan 7.1). **j** Immunohistochemistry staining of NFIB in CRC tissues and normal colorectal mucosa from THPA data. **k** Difference in NFIB staining score in CRC tissues (n = 11) and normal tissues (n = 2) from THPA data. **l** qRT-PCR for expression of NFIB in 24 pairs of CRC and normal colorectal tissues. **m** qRT-PCR for expression of NFIB in HCT-8 or SW480 cells after transfection of si-circ_0026416 or NC. **n** Schematic illustration of NFIB 3′UTR pmirGLO luciferase reporter vectors. **o** Luciferase activity of WT or MUT pmirGLO-NFIB 3′UTR after transfection with miR-346 mimics or NC in HCT-8 and SW480 cells. **p**–**s** Western blot assays showed that overexpression of hsa_circ_0026416 promotes protein expression of NFIB, while knockdown of hsa_circ_0026416 inhibits NFIB expression in HCT-8 and SW480 cells. Data are presented as means ± SD of three independent experiments. ANM = adjacent normal mucosa, TCGA = The Cancer Genome Atlas, THPA = The Human Protein Atlas, NC = negative control. *P < 0.05, **P < 0.01, ***P < 0.001

Next, taking into consideration that hsa_circ_0026416 was present primarily in the cytoplasm and many circRNAs have been proven to act as miRNA sponges, we next explored the capability of hsa_circ_0026416 to bind miRNAs. RegRNA2.0 and CircInteractome were used to predict miRNA response elements (MREs) harboured by hsa_circ_0026416. Among all predicted miRNAs, we selected the top 12 as candidate miRNAs (Fig. 4a). Among them, miR-346, miR-1184, and miR-1205 overlapped between the two databases (Fig. 4b).

MiR-346, the most downregulated by the ratio of Firefly and Renilla after transfecting pmirGLO-circ_0026416 and miRNA mimics, was chose for further analysis (Fig. 4c). Then, we utilized The Cancer Genome Atlas (TCGA) sequencing data to verify miR-346 expression in human CRC tissues (n = 601) compared to normal colorectal tissues (n = 11) (Fig. 4d) and performed qRT-PCR in 24 pairs of CRC and normal colorectal tissues to further prove the consequence of TCGA. The results both indicated that the expression of miR-346 in CRC tissues is downregulated compared to normal colorectal tissues (Fig. 4e). We further transfected si-circ_0026416 or a negative control into HCT-8 and SW480 cells using qRT-PCR to detect expression of miR-346. qRT-PCR indicated that knock down of hsa_circ_0026416 obviously increased miR-346 expression in HCT-8 and SW480 cells (Fig. 4f). Next, luciferase reporter assays were employed to ascertain whether miR-346 directly targets hsa_circ_0026416. We constructed two pmirGLO vectors, one containing the full length hsa_circ_0026416 (wild type, WT) and the other one with the miR-346-binding site mutated (MUT) (Fig. 4g). A conspicuous decrease in luciferase reporter activity was observed in HEK293T cells co-transfected with miR-346 mimics and hsa_circ_0026416 (WT), but not with the MUT vector (Fig. 4h). These results demonstrate that hsa_circ_0026416 acts as a sponge for miR-346.

MiRNAs repress transcription or induce target mRNA degradation by binding to the 3′UTR of target mRNAs [30]. To identify potential target genes of miR-346, four miRNA target gene databases (miRDB, miRWalk, starBase v3.0, TargetScan 7.1) were utilized to predict miR-346 direct target genes, and 32 genes appeared in all four databases (Fig. 4i). Among them, NFIB, which was predicted with a high score and is upregulated in human colorectal cancer as a well-known oncogene [31], was confirmed as the most likely gene directly targeted by miR-346. Next, we used The Human Protein Atlas (THPA, https://www.proteinatlas.org) proteomic data to verify the expression of NFIB in human CRC tissue compared to normal colorectal tissue (Fig. 4j). Immunohistochemistry results of tumour and normal tissues were scored as described in the Materials and Methods, and results indicated that expression of NFIB in CRC tissue was higher than in normal tissue (Fig. 4k). Next, using qRT-PCR to detect the expression of NFIB in 24 pairs of CRC and normal colorectal tissues, we obtained the same consequence (Fig. 4l). Meanwhile, Yangyang Li et al.[32] reported that miR-346 directly targets NFIB to inhibit the growth of glioma cells. In order to verify whether miR-346 could directly target NFIB in CRC cells, we constructed two pmirGLO vectors, one containing the 3′UTR of NFIB (wild type, WT) and the other one with the miR-346-binding site mutated (MUT) (Fig. 4n). A dramatic decrease in luciferase reporter activity was observed in both HCT-8 and SW480 cells co-transfected with miR-346 mimics and NFIB 3′UTR (WT), but not with the MUT vector (Fig. 4o). Next, using qRT-PCR, we discovered that expression of NFIB was decreased in response to hsa_circ_0026416 knockdown and was reduced by miR-346 mimics as well (Fig. 4m). Meanwhile, western blot showed that expression of NFIB was increased when hsa_circ_0026416 was overexpressed, whereas knockdown of hsa_circ_0026416 inhibited NFIB expression (Fig. 4p–s). Consequently, these findings verified the hsa_circ_0026416/miR-346/NFIB ceRNA network in CRC.

### Hsa_circ_0026416 facilitates CRC progression through a ceRNA network in vitro.

**Fig. 5:**
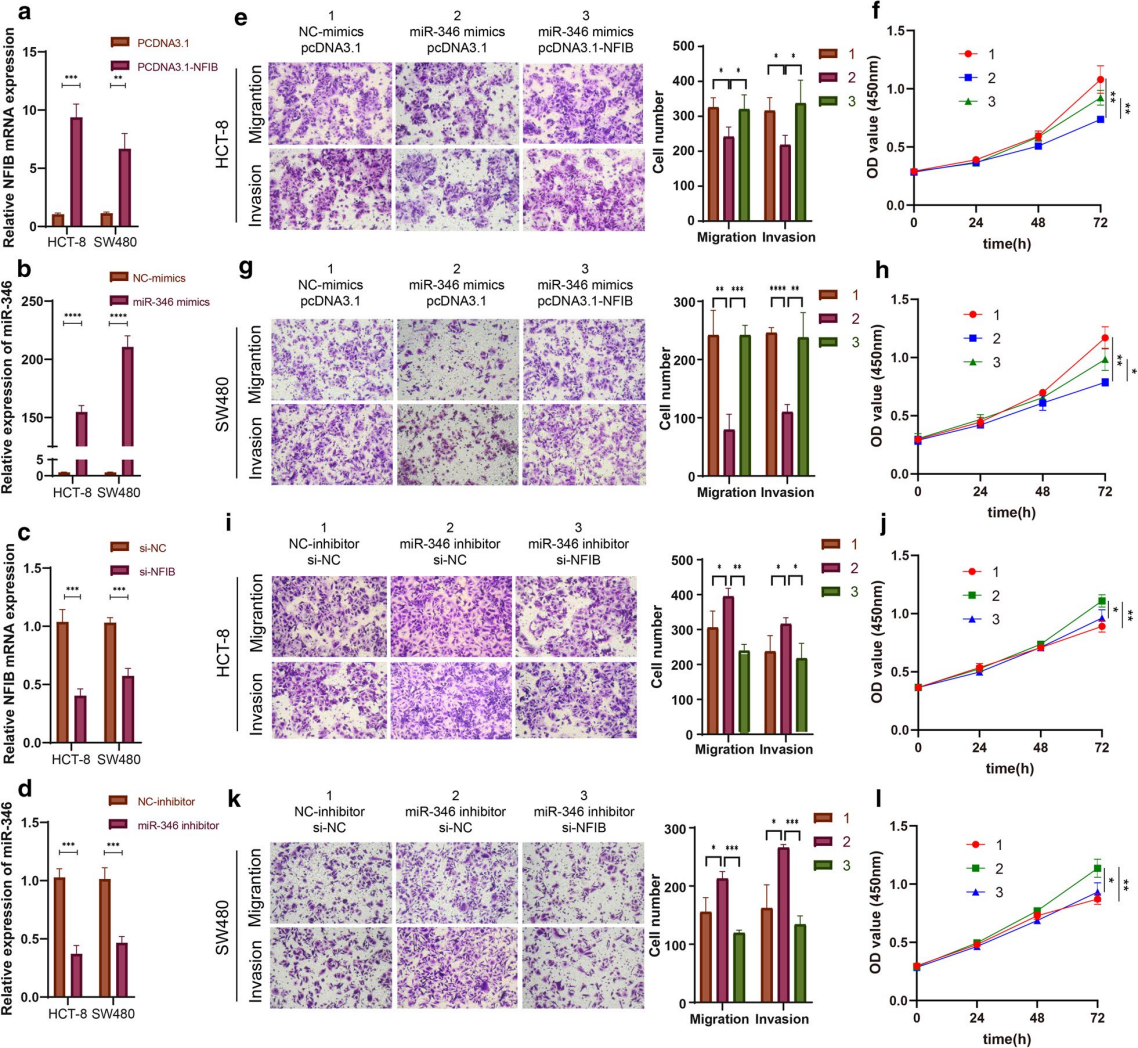
Hsa_circ_0026416 promotes CRC progression by a ceRNA pattern in vitro. **a**–**d** qRT-PCR showing that NFIB and miR-346 are upregulated in HCT-8 and SW480 cells after pcDNA3.1-NFIB, and miR-346 mimics transfection also downregulated expression in HCT-8 and SW480 cells after si-NFIB and miR-346 inhibitor transfection. **e**–**h** Transwell and CCK-8 assays indicating that pcDNA3.1-NFIB rescues the inhibitory function of miR-346 mimics on migratory, invasive and proliferative activity in HCT-8 and SW480 cells. **i**–**l** Transwell and CCK-8 assays indicating that si-NFIB rescues the enhanced function of miR-346 inhibitor on migratory, invasive and proliferative activity of HCT-8 and SW480 cells. Data are presented as means ± SD of three independent experiments. *P < 0.05, **P < 0.01, *** or ****P < 0.001

To assess whether hsa_circ_0026416 exerts biological functions in CRC through a ceRNA network, rescue experiments were performed. First, miR-346 and NFIB were overexpressed by transfection with miR-346 mimics and NFIB overexpression plasmids. Meanwhile, miR-346 and NFIB were knocked down by transfection with a miR-346 inhibitor and a small interfering RNA (si-NFIB) targeting NFIB. qRT-PCR revealed that NFIB and miR-346 were upregulated in HCT-8 and SW480 cells in response to pcDNA3.1-NFIB and miR-346 mimics co-transfection, while si-NFIB and miR-346 inhibitor co-transfection downregulated expression of NFIB and miR-346 in HCT-8 and SW480 cells (Fig. 5a–d). As anticipated, the decreased migratory, invasive and proliferative abilities induced by miR-346 mimics were rescued by co-transfecting pcDNA3.1-NFIB in HCT-8 and SW480 cells (Fig. 5e–h). These results are consistent with the above findings that the increased migratory, invasive and proliferative capacities induced by miR-346 inhibitor were regained through co-transfecting si-NFIB in HCT-8 and SW480 cells (Fig. 5i–l). These results indicated that hsa_circ_0026416 promotes CRC progression in vitro through the hsa_circ_0026416/miR-346/NFIB ceRNA network.

### Downregulation of hsa_circ_0026416 inhibits CRC proliferation in vivo

**Fig. 6:**
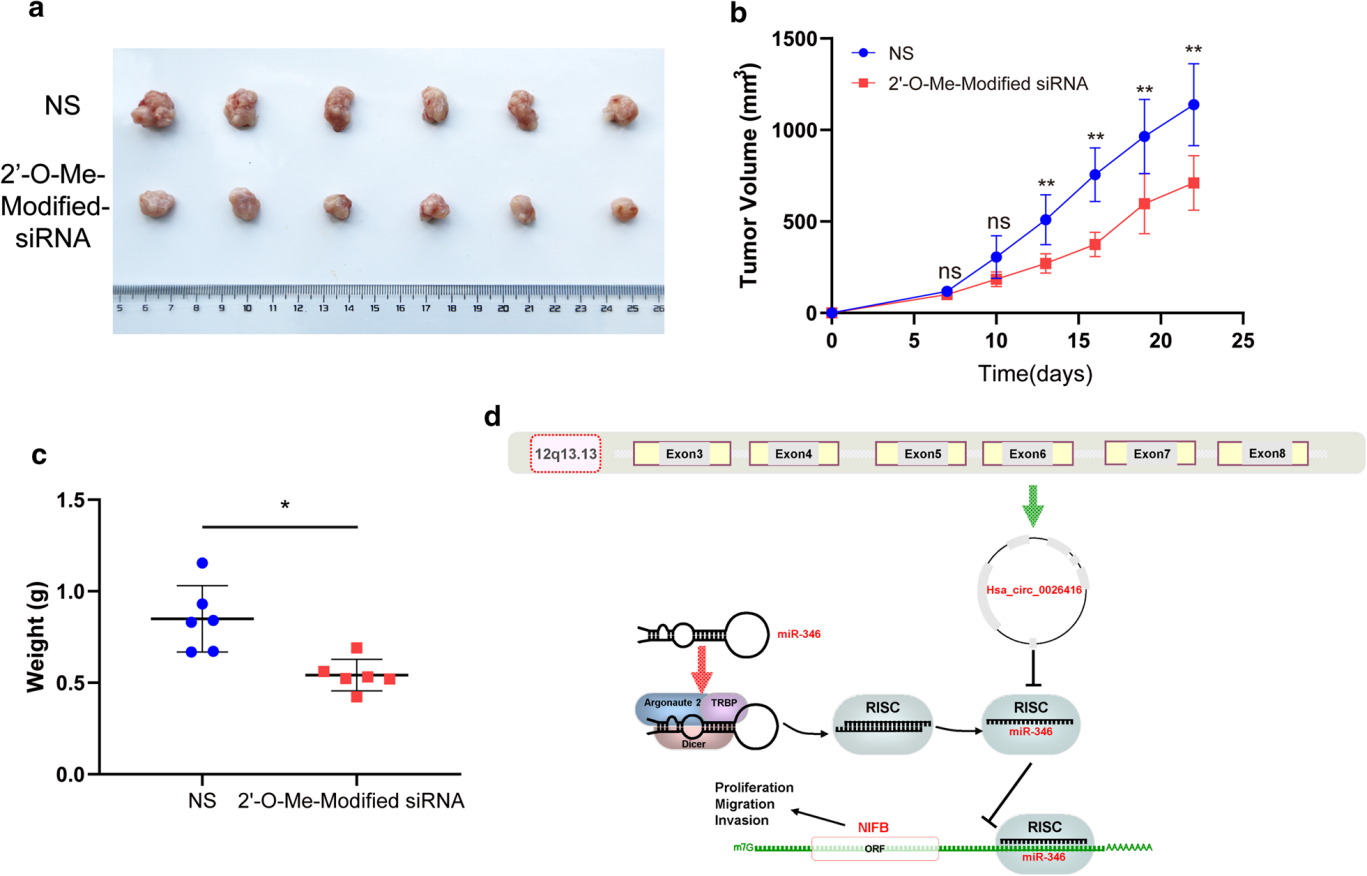
Downregulation of hsa_circ_0026416 inhibits CRC proliferation in vivo. **a** The size of xenograft tumours in the 2′-O-Me-Modified-siRNA group was significantly smaller than in the NS group. **b** The growth curve of xenograft tumours in the2′-O-Me-Modified-siRNA group was slower than the NS group. **c** The weight of xenograft tumours in the 2′-O-Me-Modified-siRNA group was reduced compared to the NS group. **d** Schematic illustration of hsa_circ_0026416 regulating the miR-346/NFIB axis in CRC. Data are presented as means ± SD of at least three independent experiments. 2′-O-Me-Modified-siRNA represents 2′-O-Me-Modified-si-circ_0026416, NS = normal saline, *P < 0.05, **P < 0.01

To further explore the tumour-promoting effect of hsa_circ_0026416 in vivo, we purchased stable small interfering RNA (2′-O-Me-Modified-si-circ_0026416) for intratumoral injection. Compared to the NS (normal saline) group, we discovered that mean tumour volume and weight in the 2′-O-Me-Modified-si-circ_0026416 group was markedly reduced (Fig. 6a–c), proving that targeted inhibition of hsa_circ_0026416 effectively inhibits tumour proliferation in vivo.

## Discussion

Although proteins are the basis of life-sustaining activities, more than 80% of the human genome is actively transcribed into RNA, while less than 3% of the human genome encodes translated proteins [33–35]. RNA molecules that are not translated into proteins are called non-coding RNAs (ncRNAs). In recent years, massive studies have indicated that ncRNAs play a crucial role in cell biology and cancer. At present, circRNAs, members of the ncRNA family, are considered to be rising stars in the study of tumorigenesis and development.

Circular RNA, which exists in the cytoplasm of eukaryotic cells, was first reported in 1979 [36]. Benefiting from the rapid advancements in high-throughput RNA sequencing, increasing circRNAs are being discovered and identified as playing vital roles in tumour progression. CircRNAs have distinct functions in different cellular locations. Cytoplasmic circRNAs have a common recognition mechanism that occurs through base pairing of the complementary region between circRNAs and their target RNA sequence.

This constitutes an intricate RNA-RNA network. For example, (i) circRNAs can interact with RNA binding proteins, such as circ-Foxo3, of which there are many. By interacting with CDK2 and p21, circRNA inhibits the cell cycle and block the transition from G1 to S phase [37]. (ii) CircRNAs can modulate the stability of mRNAs, such as circ-RasGEF1B, which strengthens the stability of ICAM-1 mRNA in mouse macrophages [38] and so on. Among them, a classical pattern known as ceRNA drew our attention. In this co-regulation pattern, circRNAs act as miRNA sponges to competitively absorb miRNAs and protect target mRNAs from suppression [39]. The first observation of a circRNA acting as a miRNA sponge was CDR1as, which has more than 70 conserved binding sites for miRNA-7 [40].

In our research, hsa_circ_0026416 was identified as an upregulated circRNA with the largest fold change (logFC = 3.70) via analysing expression profiling by high throughput sequencing from the GEO dataset (GSE77661). Hsa_circ_0026416 was upregulated in CRC tissues, plasma, and HCT-8 and SW480 cells, conveying a poor prognosis in CRC patients. In addition, our data revealed compared to CEA, CA19-9 and CA724 levels, hsa_circ_0026416 levels in plasma may represent a more promising diagnostic marker for CRC. Hsa_circ_0026416 promotes CRC cell proliferation, migration and invasion both in vitro and in vivo by competitively absorbing miR-346 and upregulating its target gene, NFIB. As previous studies have shown, the majority of circRNAs act as miRNA sponges or competitive endogenous RNAs (ceRNAs) to regulate the expression of target genes [41]. MiRNAs can combine to the matched 3′-UTRs of mRNAs, and circRNAs also include miRNA target sites. By competing for miRNAs, circRNAs indirectly regulate the translation of target mRNAs. However, circRNAs have a variety of functions, including interacting with RNA binding proteins to regulate tumour progression. This implies that hsa_circ_0026416 may also participate in additional regulatory circuits apart from the hsa_circ_0026416/miR-346/NFIB axis. This is a limitation of our research, which needs further investigation.

MiR-346, which functions as a tumour suppressor [42], was contained in both databases and was the highest-ranked miRNA. Luciferase reporter assay identified that miR-346 binds with hsa_circ_0026416. The Cancer Genome Atlas (TCGA) sequencing data indicated that miR-346 was downregulated in CRC tissues and qRT-PCR experiment in 24 pairs of CRC and normal colorectal tissues further validated the consequence of TCGA. Then, we utilized four online bioinformatics prediction databases to predict potential target genes of miR-346. NFIB appeared in all four databases and has been reported as a target gene of miR-346 in glioma [32]. Moreover, NFIB was identified as an oncogene in CRC [31, 43]. THPA database and qRT-PCR experiment both demonstrated that NFIB was upregulated in CRC. Afterwards, luciferase reporter assay further identified that miR-346 binds with NFIB. In addition, western blot indicated that knocking down hsa_circ_0026416 expression downregulated the expression of NFIB, and increasing the expression of hsa_circ_0026416 upregulated NFIB expression. These results further proved that hsa_circ_0026416 acts as a competing endogenous RNA (ceRNA) for miR-346 and upregulates expression of NFIB, further promoting proliferation and migration in CRC (Fig. 6d).

Our data indicate that hsa_circ_0026416 can be considered a potential biomarker for the diagnosis of CRC. Its expression was tremendously upregulated in CRC tissues and plasma, and its AUC value of 0.767 was higher than that of CEA (0.670), CA19-9 (0.592) and CA72-4 (0.575). In vivo experiments further revealed that targeted inhibition of hsa_circ_0026416 slowed tumour growth. Taken together, these factors all indicate that hsa_circ_0026416 is an excellent biomarker for the diagnosis of CRC and a novel target for the treatment of CRC.

In summary, our study demonstrated that hsa_circ_0026416 is upregulated in CRC tissues and CRC patient plasma. Univariate and multivariate Cox regression analysis both revealed that.

high hsa_circ_0026416 expression was the indicative of poor prognosis. Functionally, hsa_circ_0026416 promotes CRC cell proliferation, migration and invasion both in vitro and in vivo. Mechanistically, hsa_circ_0026416 acts as a ceRNA for miR-346 to upregulate NFIB and promote tumour progression (Fig. 6d). Our results indicate that hsa_circ_0026416 plays a crucial role in CRC progression and may represent an important and promising biomarker for CRC patient diagnosis and targeted therapy.

## Conclusions

Our research proves that hsa_circ_0026416 is upregulated in CRC patient tissues and plasma and high expression of hsa_circ_0026416 lead to poor prognosis in CRC patients. Hsa_circ_0026416 promotes CRC cell proliferation, migration and invasion via the miR-346/NFIB axis in vitro and in vivo. Therefore, hsa_circ_0026416 may represent a potential biomarker for diagnosis and therapeutic targeting.

## Supplementary information

**Additional file 1: Table S1.** Primers and Oligonucleotides sequences.

## Data Availability

All data generated or analysed during this study are included in the published article and are available in Gene Expression Omnibus (GEO), circBase, The Human Protein Atlas, RegRNA2.0, CircInteractome, miRDB, miRWalk, starBase v3.0 and TargetScan 7.1. (https://www.ncbi.nlm.nih.gov/geo/, https://circbase.org/, https://www.proteinatlas.org, https://regrna2.mbc.nctu.edu.tw/, https://circinteractome.nia.nih.gov/, https://mirdb.org/, https://mirwalk.umm.uni-heidelberg.de/, https://starbase.sysu.edu.cn/, https://www.targetscan.org/vert_71/).

## Abbreviations

CRC: Colorectal cancer
BC: Bladder cancer
ANM: Adjacent normal mucosa
OS: Overall survival
FBS: Fetal bovine serum
siRNA: Small interfering RNA
NC: Negative control
CCK-8: Cell counting kit-8
OD: Optical density
ROC: Receiver operating characteristic curve
AUC: Area under curve
TCGA: The Cancer Genomic Atlas
THPA: The Human Protein Atlas
