# Supplementary material for: Hsa_circ_0026416 promotes proliferation and migration in colorectal cancer via miR-346/NFIB axis
Source: Cancer Cell Int. 2020 Oct 12;20:494. doi: 10.1186/s12935-020-01593-1 (PMC7549246; doi:10.1186/s12935-020-01593-1)
Supplement: Supplementary file 1 — Additional file 1: Table S1. Primers and Oligonucleotides sequences. [file 12935_2020_1593_MOESM1_ESM.docx]

**Table S1 Primers and Oligonucleotides sequences**

| Variable | Sequence (5'-3') |
| --- | --- |
| Primers |  |
| U6 Forward | CTCGCTTCGGCAGCACA |
| U6 Reverse | AACGCTTCACGAATTTGCGT |
| GAPDH Forward | GGCCTCCAAGGAGTAAGACC |
| GAPDH Reverse | AGGGGAGATTCAGTGTGGTG |
| Hsa_circ_0026416 Forward | CACCAAGACTGTGAGGCAGA |
| Hsa_circ_0026416 Reverse | TTCATTCTCTGCTGCTGTGC |
| miR-346 Forward | ACACTCCAGCTGGGTGTCTGCCTGAGTGCCT |
| miR-346 Reverse | CTCAACTGGTGTCGTGGAGTCGGCAATTCAGTTGAGAGAGGCAG |
| NFIB Forward | TGAGGCAGCTTCACCTACAG |
| NFIB Reverse | AGGATGGGTCTCTTGGGCTTA |
| siRNAs |  |
| NC | GCGACGAUCUGCCUAAGAUdTdT |
|  | AUCUUAGGCAGAUCGUCGCdTdT |
| si-circRNA-1# | AACGUCUAUAUGAGGAUGATT |
|  | UCAUCCUCAUAUAGACGUUTT |
| si-circRNA-2# | ACAAGUCAACGUCUAUAUGTT |
|  | CAUAUAGACGUUGACUUGUTT |
| si-NC | UUCUCCGAACGUGUCACGUTT |
|  | ACGUGACACGUUCGGAGAATT |
| si-NFIB | AAGCCACAAUGAUCCUGCCAAGAAU |
|  | GUUUUAAGGCGAAGUCAUGAUGUAU |
| miR-346 mimics and inhibitor |  |
| miR-346 mimics | UGUCUGCCCGCAUGCCUGCCUCU |
|  | AGGCAGGCAUGCGGGCAGACAUU |
| NC-mimics | UUCUCCGAACGUGUCACGUTT |
|  | ACGUGACACGUUCGGAGAATT |
| miR-346 inhibitor | UGUCUGCCCGCAUGCCUGCCUCU |
| NC-inhibitor | UUGUACUACACAAAAGUACUG |

**Abbreviations:** GAPDH: glyceraldehyde 3-phosphate dehydrogenase; NFIB: Nuclear Factor I B; NC: negative control
